# Divergence in Elevation Diversity Patterns of Geckos on Two Mountains in the Hainan Tropical Rainforest National Park

**DOI:** 10.3390/ani15162410

**Published:** 2025-08-17

**Authors:** Yuting Tan, Zhixue Lin, Fanrong Xiao, Hongmin Yu

**Affiliations:** Ministry of Education Key Laboratory for Ecology of Tropical Islands, Key Laboratory of Tropical Animal and Plant Ecology of Hainan Province, College of Life Sciences, Hainan Normal University, Haikou 571158, China; 2023222040145@stu.scu.edu.cn (Y.T.); 202312071300007@hainnu.edu.cn (Z.L.); yuhongmin812@163.com (H.Y.)

**Keywords:** Gekkonidae, altitudinal gradient, Shannon–Wiener diversity index, abundance

## Abstract

Investigating altitudinal distribution patterns of species richness represents a fundamental research objective in biogeography and community ecology. Hainan Island has tropical rainforests ranging from sea level to >1800 m a.s.l., with various animal species, including reptiles such as geckos. Information on the altitudinal distribution patterns of animal diversity on Hainan Island is limited. Thus, from October 2020 to June 2023, we surveyed Gekkonidae species on Diaoluo Mountain and Jianfeng Ridge in the Hainan Tropical Rainforest National Park using a line transect method. The two study sites were divided into seven altitudinal zones at intervals of 150 m from 31 to 1080 m a.s.l. We tested correlations between abundance and species diversity indices and altitude. Five gecko species were identified. The endemic *Gekko similignum* mainly occurred at high-altitude areas on both mountains, whereas *Hemidactylus frenatus* occupied low-altitude areas. *Gehyra mutilata* had the lowest abundance among all species at all altitudes. Diaoluo Mountain exhibited a higher species diversity and abundance than Jianfeng Ridge. Geckos on Diaoluo Mountain were mainly distributed between 31 and 920 m a.s.l., presenting a bimodal distribution, with peaks appearing in altitudinal zones II (181–330 m a.s.l.) and VI (781–930 m a.s.l.). The gecko distribution on Jianfeng Ridge ranged from 31 to 948 m a.s.l., presenting a unimodal distribution, with a peak in altitudinal zone V (631–780 m a.s.l.).

## Simple Summary

Understanding how geckos are distributed across mountain elevations may inform efforts to protect tropical ecosystems. In Hainan Island’s rainforests, we studied five gecko species inhabiting two mountains (Diaoluo and Jianfeng) from 2020 to 2023. The key findings show that the endemic *Gekko similignum* thrives at higher elevations, whereas *Hemidactylus frenatus* prefers lower areas. More geckos were observed on Diaoluo Mountain, with peaks at mid (181–330 m a.s.l.) and high elevations (781–930 m a.s.l.), showing a bimodal pattern. Jianfeng Ridge had fewer geckos concentrated at mid-levels (631–780 m a.s.l.), demonstrating a typical unimodal trend.

## 1. Introduction

Mountainous regions harbour a disproportionate number of species relative to their geographic area [1], and many global biodiversity hotspots are located within such areas [2,3]. Regarding the general mechanisms underlying variations in species diversity, elevational gradients have been recognised as useful microcosms of broader species richness patterns [4,5]. Ecological factors and conditions, such as temperature, precipitation, and irradiation, change with altitude, thus producing considerable gradient effects [6,7]. Ecological factors along altitudinal gradients change 1000-fold faster than those along latitudinal gradients [8]. Therefore, mountains serve as natural laboratories that provide unique opportunities for testing ecological theories.

The altitudinal distribution patterns of animal and plant global diversity include five main types: increasing, decreasing, unimodal, low plateau, and unclear patterns [9]. With regard to animals, most invertebrates (55%) and vertebrates (64%) show unimodal patterns [2]. In previous studies, reptile species diversity exhibited four main patterns: decreasing (54%), low plateau (21%), unimodal (21%), and low plateau with a mid-elevation peak (4%) [10]. The decreasing pattern is more common among reptiles, as observed in the Australian Alps and the southwestern Ghats of India [11,12]. With regard to geckos, previous studies have confirmed either a decreasing or a low-plateau pattern [13,14,15].

The altitudinal distribution patterns of ectothermic reptiles are influenced by climatic factors, such as temperature, and anthropogenic factors. For instance, unimodal distribution patterns are frequently caused by more intense human disturbance in low-altitude areas [2]. Furthermore, for some gecko species that prefer human settlements [14,16], the presence or absence of artificial structures may significantly affect their distribution.

Information on the distribution of reptile diversity considering elevation in the tropical and subtropical areas of China is scarce [17]. Hainan Island is in the tropical region of China, with mountains of up to 1867 m a.s.l.; it is ideal for studying animal distribution patterns. However, few studies on Hainan Island examined changes in animal diversity over altitudinal gradients, and only a few animal diversity surveys have been conducted [18,19,20]. Six species from three Gekkonidae genera have been observed on Hainan Island [21]; however, ecological studies have focused on their reproductive ecology and biogeography [22,23], whereas few studies have examined their altitudinal diversity patterns.

Diaoluo Mountain (E 109°40′41.5″–110°4′42.3″, N 18°38′58.9″–18°51′0.1″) and Jianfeng Ridge (E 108°44′32.7″–109°4′5.9″, N 18°34′59.7″–18°52′46.8″) in the southeast and southwest of Hainan Island in the Hainan Tropical Rainforest National Park, respectively, are approximately at the same latitude; both have altitudes of >1400 m a.s.l. Diaoluo Mountain and Jianfeng Ridge receive a high average annual rainfall, have high evaporation rates, distinct dry and wet seasons, and rainfall characterised by monsoons and typhoons. However, owing to the mountain orientation and monsoons, the two areas differ in terms of temperature and rainfall [24]. The average annual temperature of Jianfeng Ridge tropical forest area is 0.4 °C higher than that of Diaoluo Mountain area, with the hottest and coldest months 0.5 and 2.7 °C warmer than those of Diaoluo Mountain, respectively. The annual rainfall on Jianfeng Ridge was approximately 500 mm lower than that in Diaoluo Mountain, with a less effective rainfall and a longer dry season. Global-scale studies have demonstrated significant hemispheric and latitudinal variations in elevational diversity patterns [2], yet potential differences among mountain ranges at the same latitude have received comparatively less attention. In this study, we collected data on species richness, abundance, and elevation to assess the elevational diversity and abundance pattern of gecko species on Diaoluo Mountain and Jianfeng Ridge. We hypothesised that the elevational distribution patterns of species diversity would differ between Diaoluo Mountain and Jianfeng Ridge because of the significant climatic differences between these two locations [24]. We tested correlations between altitude and gecko abundance and diversity, and our results improve our understanding of the correlation between animal diversity and elevation on Hainan Island.

## 2. Materials and Methods

### 2.1. Line Transect Design

**Figure 1:**
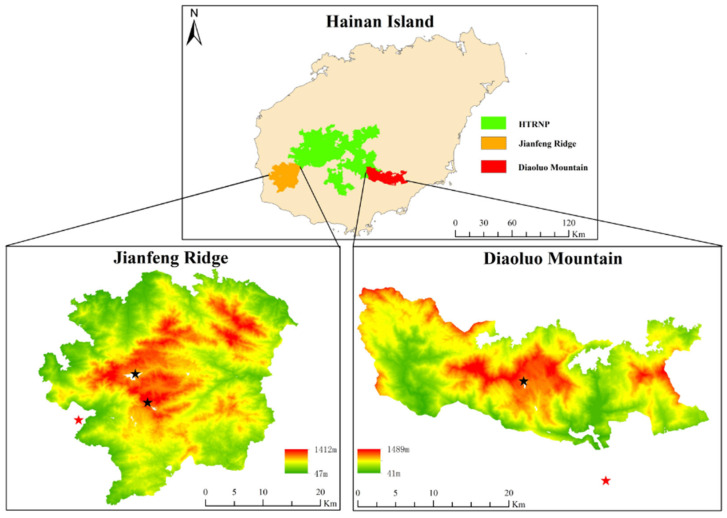
Location and elevation of Jianfeng Ridge and Diaoluo Mountain in the Hainan Tropical Rainforest National Park (HTRNP), Hainan Island, China. The red and black stars indicate the beginning and end of the line transects, respectively.

We established line transects along the Jianfeng Ridge and Diaoluo Mountain roads because of the predominant distribution of geckos along iron guardrails on the uphill side of these roads [23]. We included segments before ascending and after reaching the end of the road in the transects in order to cover multiple elevation gradients; these transitioned to wooden boardwalks or stone steps after the road ended. Diaoluo Mountain had one transect that was 26 km long. Jianfeng Ridge, with two uphill roads, had two transects that were 15 km and 18 km long (Figure 1). The starting elevation of both transects was 31 m a.s.l. The endpoint elevations for the line transects on Diaoluo Mountain and Jianfeng Ridge were 1032 m and 1107 m a.s.l., respectively (Figure 1).

### 2.2. Species Identification and Individual Counting

We conducted three surveys in October 2020, July 2022, and June 2023 using line transect sampling along the roadsides of Diaoluo Mountain and Jianfeng Ridge. Each survey was conducted for 3 days in the mornings (7:30–10:30) and evenings (19:00–21:00). Each survey team had four members—two conducted searches, while two recorded data. The team maintained a relative walking speed of 1.5–2 km/h to keep the intensity of the search effort consistent for each line transect. Visual encounter surveys were conducted to identify gecko species within the survey area, including live specimens and carcasses. The identification of Gekkonidae primarily followed references such as The Amphibia and Reptilia Fauna of Hainan [21] and the taxonomic literature [25,26]. All individuals were released at their respective capture sites after identification.

### 2.3. Altitude Segmentation

The temperature is expected to decrease by approximately 1 °C with each increase of 150 m in altitude [8]. Thus, the altitude range was divided into seven altitudinal zones, considering every 150 m increase in altitude for Diaoluo Mountain and Jianfeng Ridge, respectively. These zones were defined as altitudinal zones I (31–180 m a.s.l.), II (181–330 m a.s.l.), III (331–480 m a.s.l.), IV (481–630 m a.s.l.), V (631–780 m a.s.l.), VI (781–930 m a.s.l.), and VII (931–1080 m a.s.l.).

### 2.4. Calculation of Abundance and Species Diversity Indices per Site

The abundance is the cumulative number of individuals per species for the three surveys combined. Shannon–Wiener and Simpson’s diversity indices are metrics used in the study of elevational diversity–distribution patterns [27] and were used in the present study to calculate gecko species diversity using the following formula:

Shannon–Wiener Index (H):

(1) $$H=-\sum_{i=1}^{s}(\ln P_{i})(P_{i})$$

Simpson’s Diversity Index (D):

(2) $$D=1-\sum_{i=1}^{s}(P_{i}{)}^{2}$$

where Pi is the relative frequency of species i in the community; S is the number of species in the community.

### 2.5. Statistical Analyses

The number of species and subsequent abundances per each altitude zone were recorded in a spreadsheet. Data from the two line transects at the same altitude on Jianfeng Ridge were merged into one dataset. As geckos are mostly active at dawn and dusk, we chose to investigate during these two periods. However, due to the limited data, we did not separately compare the data from the two periods in both mountains. Line graphs were constructed using the raw data to visualise changes in the abundances of individual species considering altitude. A polynomial regression fitting analysis was performed to fit the variation in species diversity along the altitudinal gradient. The first to fifth polynomial regression fittings were performed. An overfitting trend appeared in the fourth and fifth polynomial regression fits, whereas the fitting effect of the cubic polynomial regression was significant. Therefore, a cubic polynomial regression was chosen. The fundamental model is expressed as F(X) = aX^3^ + bX^2^ + cX + d. All statistical analyses and plots were produced using the ggplot2 package on R software version 4.3.3 [28].

## 3. Results

### 3.1. Correlation Between Species Abundance and Altitude

**Figure 2:**
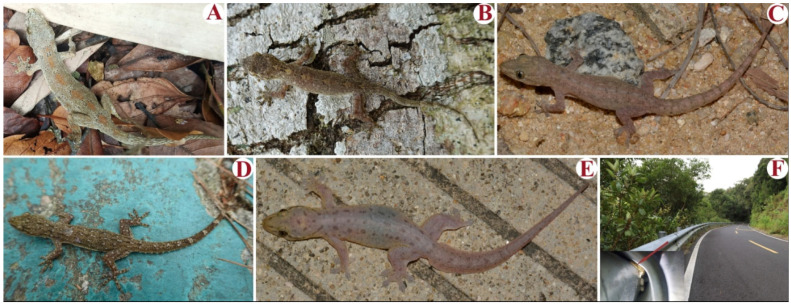
Five gecko species and their habitats. (**A**): *Gekko similignum*; (**B**): *Hemidactylus bowringii*; (**C**): *H. frenatus*; (**D**): *H. garnotii*; (**E**): *Gehyra mutilata*; (**F**): Iron guardrail and its adjacent habitat (the lower right photograph shows a *G. similignum* nest site and eggs).

We recorded 799 geckos on Diaoluo Mountain, belonging to five species from three genera—*Gekko similignum*, *Hemidactylus bowringii*, *H*. *frenatus*, *H*. *garnotii*, and *Gehyra mutilata* (Table 1 and Figure 2). We recorded 465 geckos on Jianfeng Ridge, belonging to four species, i.e., *Gekko similignum*, *H. bowringii*, *H. frenatus*, and *H. garnotii* (Table 1). Except for *H. frenatus*, the abundance of each species was greater on Diaoluo Mountain than on Jianfeng Ridge (Table 1).

**Figure 3:**
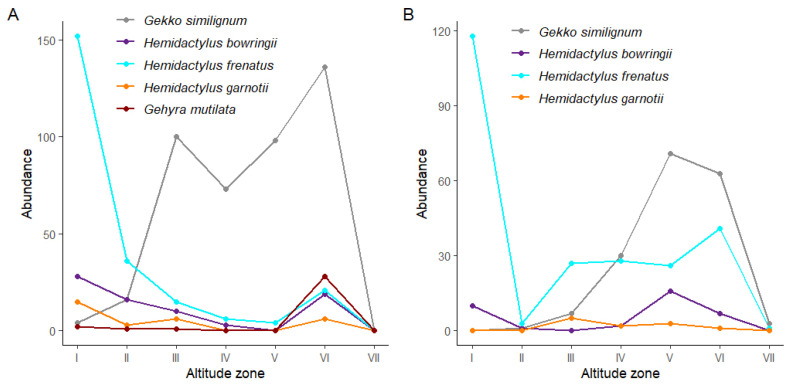
Altitudinal variation in gecko abundance per species on Diaoluo Mountain (**A**) and Jianfeng Ridge (**B**). Altitude zones I (31–180 m a.s.l.), II (181–330 m a.s.l.), III (331–480 m a.s.l.), IV (481–630 m a.s.l.), V (631–780 m a.s.l.), VI (781–930 m a.s.l.), and VII (931–1080 m a.s.l.).

Geckos were recorded on both sites along all the altitudinal zones up to 930 m a.s.l., with some individuals found in zone VII only on Jianfeng Ridge (Table 1). On Diaoluo Mountain and Jianfeng Ridge, the highest abundance of *Gekko similignum* was at relatively high altitudes, whereas that of *Hemidactylus garnotii* was observed at low altitudes (Figure 3). The abundance of *H. bowringii* and *H. garnotii* was relatively low on both Jianfeng Ridge and Diaoluo Mountain, and the change was not obvious with the increase in altitude (Figure 3). *Gehyra mutilata* found on Diaoluo Mountain were more abundant at high altitudes (Figure 3).

### 3.2. Altitudinal Distribution Patterns of Species Diversity

**Figure 4:**
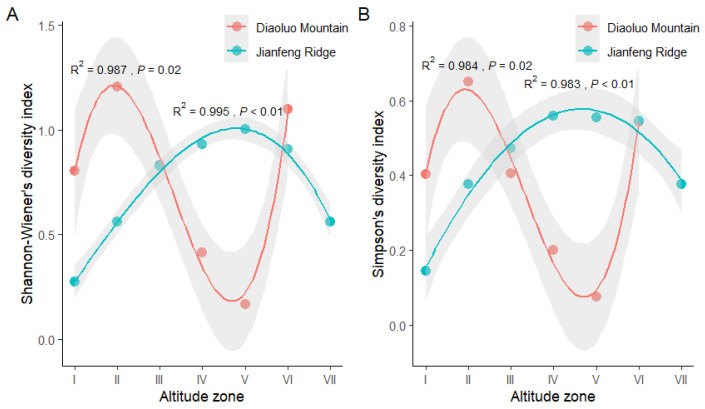
Elevation diversity pattern of Shannon–Wiener (**A**) and Simpson’s diversity index (**B**) of geckos on Diaoluo Mountain and Jianfeng Ridge. Altitude zones I (31–180 m a.s.l.), II (181–330 m a.s.l.), III (331–480 m a.s.l.), IV (481–630 m a.s.l.), V (631–780 m a.s.l.), VI (781–930 m a.s.l.), and VII (931–1080 m a.s.l.). Solid dots represent the observed species diversity, solid lines represent the species diversity predicted by the model, and grey areas represent the 95% confidence interval predicted by the model.

The Shannon–Wiener diversity index significantly fit the altitudinal gradient on Diaoluo Mountain (F(X) = 0.097X^3^ − 0.957X^2^ + 2.607X − 0.948; R^2^ = 0.987, *p* = 0.02) and Jianfeng Ridge (F(X) = −0.006X^3^ + 0.018X^2^ − 0.275X − 0.009; R^2^ = 0.995, *p* < 0.01) (Figure 4A). The diversity–distribution pattern of Gekkonidae species on Diaoluo Mountain exhibited a bimodal pattern, with peaks occurring in altitude zones II (181–330 m a.s.l.) and VI (781–930 m a.s.l.) (Figure 4A). The diversity–distribution pattern of Gekkonidae species on Jianfeng Ridge exhibited a unimodal pattern, with a peak appearing in altitude zone V (631–780 m a.s.l.) (Figure 4A). Similarly, the Simpson’s diversity index of Gekkonidae species exhibited a significant fit with the altitudinal gradient on Diaoluo Mountain (F(X) = 0.051X^3^ − 0.510X^2^ + 1.394X − 0.527; R^2^ = 0.984, *p* = 0.02) and Jianfeng Ridge (F(X) = −0.001X^3^ − 0.025X^2^ + 0.274X − 0.094; R^2^ = 0.983, *p* < 0.01) (Figure 4B). The diversity–distribution pattern was bimodal on Diaoluo Mountain, with peaks occurring in altitude zones II (181–330 m a.s.l.) and VI (781–930 m a.s.l.) (Figure 4B). Jianfeng Ridge showed a unimodal pattern with a peak in altitude zones IV (481–630 m a.s.l.) or V (631–780 m a.s.l.) (Figure 4B).

## 4. Discussion

### 4.1. Effect of Altitudinal Variation on Gecko Abundance

Considering both sampled localities in this study, five species belonging to three genera of geckos were identified. Among the six gecko species previously reported in Hainan [21], *Gekko gecko* was not found in this study. According to Shi et al. [21], the occurrence of *Gekko gecko* in the wild is improbable, as it may be a non-native species of Hainan Island. Although our current survey results are consistent with this conclusion, further in-depth research is needed to determine whether this species has historically existed or currently occurs on Hainan Island.

No geckos were found between 920 and 1032 m a.s.l. on Diaoluo Mountain and between 948 and 1107 m a.s.l. on Jianfeng Ridge, indicating that the upper altitude limit for the survival of these species was approximately 948 m a.s.l. There were two species, *Gekko similignum* and *Hemidactylus frenatus*, distributed above 930 m in altitude, whereas the other three species were distributed below 930 m. The highest peak in Hainan—Wuzhi Mountain (1867 m a.s.l.)—was not surveyed. Thus, further investigations are required to determine the upper distribution limit of these gecko species on Hainan Island.

The diversity and abundance of gecko species on Jianfeng Ridge (four species and 465 individuals) were lower than those on Diaoluo Mountain (five species and 799 individuals) in this study (Figure 4), which is consistent with the trends observed in other amphibians and reptiles [29,30]. This is probably because Diaoluo Mountain has a more tropical rainforest climate, supporting a higher species diversity and abundance. *Gehyra mutilata* may be distributed on Jianfeng Ridge, as noted by Shi et al. [21], but was not observed in the present survey because of its very low abundance in the area. This species mainly inhabits higher-altitude areas on Diaoluo Mountain but was scarce in the present study.

The trends of individual species along the altitudinal gradient were similar on both mountains. *Gekko similignum* occupied and was predominant in high-altitude areas. Conversely, *Hemidactylus frenatus* occupied and was predominant in low-altitude areas. Its abundance accounts for 65% and 48% in Altitude zone I of Diaoluo Mountain and Jianfeng Ridge, respectively. This altitudinal niche separation reduces competition between the two species.

### 4.2. Altitudinal Distribution Patterns of Species Diversity

Regardless of the diversity index used, the species diversity–altitude pattern on Diaoluo Mountain exhibited a bimodal distribution, whereas that of Jianfeng Ridge exhibited a unimodal distribution, which is inconsistent with findings from previous studies on geckos [13,15,31]. Bimodal distribution patterns have not been observed in reptiles; this is also the pattern with the smallest proportion in current global-scale research [2,32]. Although unimodal distributions are common in vertebrates (64%) [2] and reptiles [10], they were observed here for the first time in geckos, which differs from a study on lizards showing a low plateau with a mid-altitude peak [33].

The two peaks of gecko species diversity on Diaoluo Mountain were located at low (zone II) and high altitudes (zone VI). These locations are within protected areas but also feature human-made structures, representative of a moderately disturbed environment. Geckos prefer artificial structures because artificial light at such locations attracts insects, which they consume [14,16]. Furthermore, high-altitude areas have open spaces with lower forest canopy densities than mid-altitude areas [23], allowing for sufficient sunlight exposure for optimal physiological activities and reproduction, thereby offsetting the limitations imposed by low temperatures at high altitudes. We previously found that *Gekko similignum* prefer to lay eggs beside roads with moderate canopy density rather than in areas with high canopy density, as the temperature in such areas is not suitable for egg hatching [23]. Furthermore, it seems that this bimodal pattern is mostly driven by endemic *Gekko similignum* adapted to higher elevations. The first gecko diversity peak was higher than that of the second peak, indicating that temperature variations at different altitudes impact the diversity–distribution pattern of geckos on Diaoluo Mountain. Another possible explanation is that several abiotic factors related to temperature change with elevation, for example, rainfall and humidity, affect the distribution and diversity of ectotherms, but any combination of these could cause a change in gecko diversity [34].

The unimodal pattern observed on Jianfeng Ridge is attributed to high temperatures and relatively drier conditions than those on Diaoluo Mountain [24], rendering low altitudes unconducive for most gecko species. Therefore, Jianfeng Ridge did not exhibit a high gecko abundance at low altitudes, as observed on Diaoluo Mountain. Additionally, mid-elevations can act as a “thermoecological ecotone,” providing conducive conditions for a relatively large number of species, with species habituating in high- and low-elevation likely to cohabitate in these areas [35]. The fitted curves of the Shannon–Wiener and Simpson’s diversity indices revealed that species diversity on Jianfeng Ridge was relatively constant in altitude zones IV, V, and VI, as the climatic conditions within these altitude ranges were relatively stable [24]. Another possible explanation is that some low-altitude areas fall outside the national park and experience intense anthropogenic disturbances, leading to decreased species diversity and ultimately resulting in a unimodal distribution pattern [2].

## 5. Conclusions

Although Diaoluo Mountain and Jianfeng Ridge are at the same latitude on Hainan Island, their elevational diversity patterns differ. Diaoluo Mountain exhibited a bimodal pattern, whereas Jianfeng Ridge exhibited a unimodal pattern. To the best of our knowledge, this is the first observation of such a bimodal pattern in reptiles and a unimodal pattern in geckos. However, because of the limitations of our survey transects, which only covered habitats along roads, these findings require further data validation and support in future studies. Based on comprehensive investigations, future studies should also quantitatively assess the climatic factors of each elevation zone, elucidate the factors driving the spatial distribution patterns of Gekkonidae diversity on Hainan Island, and predict patterns of species migration along elevational gradients driven by climate change.

## Figures and Tables

**Table 1:** Abundance of geckos on Diaoluo Mountain (DLM) and Jianfeng Ridge (JFR) by species.

Altitude Zone	*Gekko similignum*		*Hemidactylus bowringii*		*Hemidactylus frenatus*		*Hemidactylus garnotii*		*Gehyra mutilata*		Total	
	DLM	JFR	DLM	JFR	DLM	JFR	DLM	JFR	DLM	JFR	DLM	JFR
I (31–180 m)	4	0	28	10	152	118	15	0	2	0	201	128
II (181–330 m)	16	1	16	0	36	3	3	0	1	0	72	4
III (331–480 m)	100	7	10	0	15	27	6	5	1	0	132	39
IV (481–630 m)	73	30	3	2	6	28	0	2	0	0	82	62
V (631–780 m)	98	71	0	16	4	26	0	3	0	0	102	116
VI (781–930 m)	136	63	19	7	21	41	6	1	28	0	210	112
VII (931–1080 m)	0	3	0	0	0	1	0	0	0	0	0	4
Total	427	175	76	35	234	244	30	11	32	0	799	465

## Data Availability

The datasets used during the current study are available from the corresponding author upon reasonable request.
